# Esports: The Chess of the 21st Century

**DOI:** 10.3389/fpsyg.2019.00156

**Published:** 2019-01-30

**Authors:** Matthew A. Pluss, Kyle J. M. Bennett, Andrew R. Novak, Derek Panchuk, Aaron J. Coutts, Job Fransen

**Affiliations:** ^1^Human Performance Research Centre, Faculty of Health, University of Technology Sydney, Moore Park, NSW, Australia; ^2^High Performance Department, Rugby Australia, Moore Park, NSW, Australia; ^3^Institute of Sport, Exercise and Active Living, Victoria University, Melbourne, VIC, Australia; ^4^Movement Science, Australian Institute of Sport, Bruce, ACT, Australia

**Keywords:** electronic sports, expertise, expert performance, excellence, skilled performance, video games, gaming

## Abstract

For many decades, researchers have explored the true potential of human achievement. The expertise field has come a long way since the early works of de Groot (1965) and Chase and Simon (1973). Since then, this inquiry has expanded into the areas of music, science, technology, sport, academia, and art. Despite the vast amount of research to date, the capability of study methodologies to truly capture the nature of expertise remains questionable. Some considerations include (i) the individual bias in the retrospective recall of developmental activities, (ii) the ability to develop ecologically valid tasks, and (iii) difficulties capturing the influence of confounding factors on expertise. This article proposes that expertise research in electronic sports (esports) presents an opportunity to overcome some of these considerations. Esports involves individuals or teams of players that compete in video game competitions via human-computer interaction. Advantages of applying the expert performance approach in esports include (i) developmental activities are objectively tracked and automatically logged online, (ii) the constraints of representative tasks correspond with the real-world environment of esports performance, and (iii) expertise has emerged without the influence of guided systematic training environments. Therefore, this article argues that esports research provides an ideal opportunity to further advance research on the development and assessment of human expertise.

## Introduction

Exploring the boundaries of human performance has fascinated researchers and practitioners in a range of fields and domains (Ericsson and Smith, 1991; Gagné, 2004; Williams and Ericsson, 2005; Gagné, 2013). Decades ago, de Groot (1965) and Chase and Simon (1973) investigated the complex thoughts and processes of expert chess players. Since then, the expert performance approach has been applied to sport (Starkes and Ericsson, 2003; Williams and Ericsson, 2005; Côté et al., 2007), music (Ericsson et al., 1993; Lehmann and Ericsson, 1997; Tang and Giddins, 2016), medicine (Gordon, 1988; Ericsson et al., 2007; Tang and Giddins, 2016), and art (Augustin and Leder, 2006; Mullennix and Robinet, 2018). The aim of the expert performance approach is to identify the key characteristics of an expert and understand how expertise is developed over time (Ericsson and Smith, 1991; Charness and Tuffiash, 2008). According to the expert performance approach, capturing human expertise has three distinct stages (Ericsson and Smith, 1991; Williams and Ericsson, 2005). The first stage involves capturing expert performance of a real-world environment in laboratory-testing (e.g., video/film and virtual reality) and/or field setting (e.g., match analysis and simulations). The second stage identifies the underlying mechanisms with process-tracing measures (e.g., visual search behavior, film occlusion, and verbal reports). The third stage is examining how expertise develops through practice history profiling (e.g., questionnaires, interviews, and logbooks) and learning studies (e.g., training interventions). Although the expert performance approach offers a theoretical guide to investigate expertise, considerations about the application of the approach have been raised.

Capturing the skills that define expertise within any domain is a hallmark of the expert performance approach (Ericsson and Smith, 1991; Williams and Ericsson, 2005). This becomes a challenge in domains where a clear and measurable performance outcome is lacking (Williams and Ericsson, 2005). For example, in sport, behavioral constructs (e.g., anticipation and decision-making) are difficult to assess within a controlled laboratory setting under standardized conditions (Williams and Ericsson, 2005; Afonso et al., 2012). The difficulty here is developing representative tasks that correspond with the constraints of a real-world environment. This consideration will influence the range of methodologies researchers develop to identify the key characteristics of experts (Ericsson and Smith, 1991; Williams and Ericsson, 2005). Whether distinguishing differences between skill groups or predicting superior performance, the lack of task representativeness has the potential to undermine the validity and reliability of the data. Another consideration is that expertise is largely influenced by external factors, such as talent development programs (e.g., sporting organizations or selective schools) and the guidance of a mentor (e.g., sporting coach or music teacher). Yet, researchers have difficulties capturing the interaction of these confounders towards the development of expertise (Baker et al., 2003). The interrelationship between these factors can complicate our interpretation about the nature of expertise. It has been recommended that future research should embrace new technology that can simulate the constraints of a real-world environment within a controlled laboratory setting, while also collecting accurate and reliable data (Williams and Ericsson, 2005; Boot, 2015; Boot et al., 2017). Given the recent advancements in technology, a new domain known as electronic sports (esports) has emerged. As human-computer interaction mediates esports performance, the virtual nature of esports may not be affected to the same extent by the limitations of previous expertise research. Therefore, this leading article argues that esports provides an excellent opportunity to further advance research on the development and assessment of human expertise.

## What is Esports?

Esports involve individuals and/or teams of players who compete in video game competitions through human-computer interaction. Participation in esports has increased substantially over the past decade, with an estimated population over 100 million players worldwide. To date, esports research has primarily focused on the factors that influence participation (Griffiths et al., 2003; Braun et al., 2016; Seo, 2016; Seo and Jung, 2016). Although information about player engagement is becoming clear, a scarce amount of research has investigated the factors that underlie expertise in esports. Esports consists of several categories, some of which include multiplayer online battle arena, multiplayer online role-playing, real-time strategy and first-person shooter. A common theme among these categories is that performance is typically carried out in a team-based environment, where a player’s avatar is placed in a virtual environment with the goal of eliminating their competitors or achieve an objective (e.g., capture the flag). A player must combine their perceptual-cognitive abilities (e.g., anticipation, visual search behavior, pattern recall, and decision-making) and domain-specific skills (e.g., keyboard and mouse movements) to achieve successful performance. Given esports performance is mediated by human-computer interaction, there are many inherent advantages for expertise research. Firstly, developmental activities are objectively tracked and automatically logged online, which can be used to provide a detailed report on a player’s development. Secondly, the constraints of a laboratory setting correspond with a real-world environment of esports. Thirdly, as esports has recently emerged, expertise is yet to be confounded by the influence of guided systematic training environments. The following subsections will discuss in detail the advantages of esports performance research offers to the expertise field.

## The Developmental Activities Related to Expertise

A prominent area of expertise research is aimed towards understanding how practice can lead to the attainment of expertise (Côté et al., 2007; Ward et al., 2007; Ericsson, 2014). Accurately mapping an individual’s practice history can elicit key insights about developmental milestones (Côté and Abernethy, 2012; Ford and Williams, 2012). Qualitative interviews, training study questionnaires and diary reports are commonly used methods to establish a practice history profile (Baker et al., 2003; Memmert et al., 2010; Côté and Abernethy, 2012). Across these methods, participants retrospectively recall their practice activities over their career. Given careers can span many years, careful interpretation is recommended when analyzing the information (Howard, 2011). However, this approach is influenced by an individual’s recall bias and memory recall ability. Participants generally overestimate the number of practice hours and time since recent milestones and underestimate the number of practice hours and the time since distant ones, often referred to as the telescoping effect (Kemp, 1988; Howard, 2011). This effect can compromise the validity and reliability of data collected using retrospective recall.

Longitudinal follow-up studies are a valuable alternative compared with retrospective recall. However, longitudinal data collection on human behavior can be expensive, time consuming and requires a large sample size because of poor participant retention (Patel et al., 2003). Despite the clear strengths of the design, this creates difficulties when developing practice history profiles that accurately reflect the trajectory of development. Comparatively, esports software records practice activities online which may provide a suitable medium to further explore the relationship between developmental activities and expertise. Both the quantity (e.g., total hours played, matches played, etc.) and quality of performance (e.g., outcome of match, player rating, performance rank, etc.) are automatically logged on online servers that are freely available to the public. The public repositories can establish a practice history profile, which can be updated to follow a player’s developmental trajectory throughout their career. Profiling can be performed for all skill levels of esports players, which is useful for tracking across a developmental spectrum rather than just from a dichotomous viewpoint (e.g., elite vs. non-elite) (Swann et al., 2015). Another example is tracking a players and/or teams practice activity throughout a competitive season. Researchers can examine the contribution that different practice activities have on performance. Information gained from this approach can inform coaches and practitioners with developing effective training strategies when preparing for competition. Using this approach reduces the logistical demands on data collection and data analysis, making it a cost effective and time efficient method of analyzing an individual’s career trajectory. Another advantage is that the influence of individual recall bias is negated, which improves the validity and reliability of the specific performance outcome measures of interest. While much of the data is publicly available, researchers must ensure that they comply with the legislations, rules and policies of their institution’s ethics committee and national regulations. If the data is obtained through a third-party repository, it must comply to the original data licensing structure. Further consideration is required for aspiring players under the age of 18, as their publicly available data will be collected when they are a minor. However, the minimum age requirement to participate in a prized pool tournament is 18 years of age. Lastly, in most cases players have the option to list their online profile as private to prevent access to their personal data. As such, the complex and sensitive nature of this method must be conveyed to the appropriate ethics committee for review. Collectively, esports provides a platform to quantify the developmental activities related to expertise without the limitations associated with long-term follow up studies that rely on retrospective recall.

## The Development of Ecologically Valid Tasks

A key area of expertise research is developing tasks that provide accurate and reproducible measurements that can be objectively evaluated in a controlled laboratory setting (Mann et al., 2007; Williams et al., 2011). Many studies in controlled laboratory settings use a form of technology to simulate the constraints of a real-world environment of the domain in question (Williams et al., 2002; Williams and Ericsson, 2005). However, concerns have been raised about whether such tasks indirectly measure a related function or ability rather than the specific and complex mechanisms that mediate expert performance (Williams and Ericsson, 2005; Hadlow et al., 2018). Over time researchers have continued to improve the ecologically validity of task-representative designs to closely resemble the dynamic and ever-changing nature of a real-world environment (Williams and Ericsson, 2005; Burgess et al., 2006; Mann et al., 2007). Research studies aimed at quantifying expert performance often trade-off external validity for the internal validity of a task, and vice versa. Evidentially, it remains difficult to develop task representative designs that allow participants to (re)produce the behavior’s observed in a real-world environment while maximizing the control that can be exerted over a task.

As human-computer interaction mediates esports performance, it may provide the ideal platform to investigate expertise as task representative designs resemble a real-world environment, without sacrificing internal validity. Traditional task representative designs have used technology (e.g., television screens, computer monitors, and video projector screens) with simulated responses (e.g., pressing a button or key and moving a joystick or mouse) (Hadlow et al., 2018). Despite being highly controllable, the implementation of this method instead of more externally valid tasks will inadvertently alter the perception-action coupling of a real-world environment (Kay and Kelso, 2016). However, as esports is mediated by this form of technology for both competition and training, the perception-action coupling experienced during performance can be accurately replicated in a controlled laboratory setting. Researchers can customize in-built settings within the software used in esports to develop highly controlled training interventions without sacrificing task representativeness. An example of this is assessing a range of performance-related characteristics, such as fine-motor coordination, processing ability and decision-making. Assessing a range of performance-related characteristics can highlight differences between esports categories where certain characteristics may be more necessary than others. Certain esports (e.g., League of Legends) have regular in-game changes to address game balance issues or provide new content for players. Therefore, the authors propose that researchers should state the current version of the game and provide a reference to the rules at that point of time whenever possible. In rare circumstances that major rule changes occur, these differences should be stated explicitly in-text when discussing esports studies together. Furthermore, an esports player’s behavioral response can be measured through the available hardware (e.g., keyboard and mouse responses). Esports with a clear and measurable performance outcome are more suited for performance-related research. Examples where researchers and practitioners can objectively examine a player’s performance include the HLTV rating for Counter-Strike: Global Offensive and the Kill/Death/Assist ratio for League of Legends. In terms of learning-related research, esports that allow researchers and practitioners to develop online training scenarios are more suitable. Examples where researchers and practitioners can create their own training interventions within a realistic environment include the authoring tool for Counter-Strike: Global Offensive and the practice tool for League of Legends. Hence, the virtual nature of esports performance can translate a real-world environment within a controlled laboratory setting under standardized conditions.

## The Confounding Factors That Influence Expertise

The developmental process of systematically developed expertise reflects the dynamic interaction between natural abilities, intrapersonal skills and environmental factors (Gagné, 2004; Ericsson et al., 2009; Gagné, 2009). Furthermore, catalysts (i.e., intrapersonal, environmental and chance) can either assist or hinder the developmental process (Gagné, 2004). A commonly reported catalyst that confounds the development of expertise is the influence of a guided systematic training environment (Barab and Plucker, 2002; Burgess and Naughton, 2010). Within traditional domains of expertise, individuals who demonstrate an aptitude are identified at an early age and selected to undergo a structured development program. A structured development program is a commonplace in music (i.e., music academies and conservatories), sport (i.e., sports schools and talent development programs), and education (i.e., selective schools for gifted and talented students). These programs provide individuals with high-quality resources (e.g., support staff, specialized coaching, and logistical support) to develop their natural abilities into talents, with the goal of developing excellence (Côté and Abernethy, 2012). As such, the development of expertise is confounded by the practices implemented across the many guided systematic training environments that exist (Baker et al., 2003). However, the effect a guided systematic training environment has on the development of expertise is difficult to quantify. Therefore, investigating expertise in a domain that has been exposed to these confounders to a lesser extent could provide an opportunity into the development of expertise outside of the constraints of guided systematic training environments.

There are many confounders in traditional domains of expertise, such as maturational factors, the role of coach, support from significant others and cultural factors, among others (Baker et al., 2003; Baker and Horton, 2004). The interaction between these factors underlies the likelihood of developing expertise, which is largely determined by the access to a guided systematic training environment (Baker et al., 2003). However, the emergence of esports professionalism has only recently sparked the development of specialized high-performance centers and support staff focused on developing excellence. Therefore, the existing pool of expert esports players have emerged largely without guided systematic training environments. As the professionalism of esports continues to increase, the access to publicly available data of high-level teams is becoming less accessible. Therefore, the authors encourage esports to follow traditional monitoring approaches by embedding researchers within professional teams to collect data at the highest level of competition. Additionally, the growing population of esports players offers a wealth of information about the interaction of natural abilities and intrapersonal skills. Therefore, esports is a domain that is more likely to reflect an individual’s raw abilities and skills as it is yet to be tainted by many of the confounders that complicate our understanding about the development of expertise.

## Conclusion

The purpose of this leading article was to provide rationale for why esports is the ideal domain for those with an interest in the assessment and development of human expertise. Three key advantages of applying the expert performance approach in esports were discussed in this article: (i) developmental activities are objectively tracked and automatically logged online, which can be used for a detailed report on a player’s developmental trajectory, (ii) the constraints of representative tasks correspond with the real-world environment of esports performance, which translates a real-world environment within a controlled laboratory setting under standardized conditions, and (iii) expertise has emerged without the influence of guided systematic training environments, which presents an opportunity to investigate in a domain yet to be tainted by many of the confounders that complicate our understanding about the development of expertise. As such, esports provides a window for researchers to further improve their understanding about the assessment and development of human expertise in the modern world. Therefore, the authors recommend embracing this emerging area as it may have the answers to many of the future recommendations that the expertise field continues to seek.

## Author Contributions

All authors listed have made a substantial, direct and intellectual contribution to the work, and approved it for publication.

## Conflict of Interest Statement

The authors declare that the research was conducted in the absence of any commercial or financial relationships that could be construed as a potential conflict of interest.
